# Participatory construction of an assistive technology for leprosy contact surveillance

**DOI:** 10.1590/0034-7167-2024-0488

**Published:** 2025-12-12

**Authors:** Breno Augusto Silva Duarte, Laura Maria Vidal Nogueira, Haroldo Gonçalves de Jesus, Ivaneide Leal Ataíde Rodrigues, Ingrid Bentes Lima, Raquel Gomes da Silva

**Affiliations:** I Universidade do Estado do Pará. Belém, Pará, Brazil

**Keywords:** Nursing, Primary Health Care, Contact Tracing, Leprosy, Health Technology., Enfermería, Atención Primaria de Salud, Trazado de Contacto, Lepra, Tecnología para la Salud.

## Abstract

**Objectives:**

to develop, in a participatory manner, an assistive technology to contribute to the nurses’ work process when managing leprosy contacts in Primary Health Care.

**Methods:**

this is a methodological study with a participatory interface. Data were collected between March and October 2023 at 19 Basic Health Units, with 29 nurses interviewed individually. IRAMUTEQ software was used for data analysis.

**Results:**

three thematic axes were organized, highlighting: Concepts regarding leprosy contact surveillance and decision-making; Operational difficulties in the work process; and Participatory development of assistive technology. Nurses supported the development of the technology in the form of a monitoring book, offering suggestions for improving the product for use in daily healthcare settings.

**Final Considerations:**

the participatory development process enabled the creation of an assistive technology aligned with nurses’ needs for managing and monitoring contacts in Primary Health Care.

## RESUMEN

**Objetivos:**

desarrollar, de forma participativa, una tecnología de asistencia para contribuir al proceso de trabajo del personal de enfermería que gestiona los contactos de lepra en Atención Primaria de Salud.

**Métodos:**

se trata de un estudio metodológico con una interfaz participativa. Los datos se recopilaron entre marzo y octubre de 2023 en 19 Unidades Básicas de Salud, con 29 enfermeras entrevistadas individualmente. Se utilizó el software IRAMUTEQ para el análisis de datos.

**Resultados:**

se organizaron tres ejes temáticos, destacando: conceptos sobre la vigilancia de los contactos de lepra y la toma de decisiones; dificultades operativas en el proceso de trabajo; y desarrollo participativo de tecnología de asistencia. Las enfermeras apoyaron el desarrollo de la tecnología mediante un cuaderno de seguimiento, ofreciendo sugerencias para mejorar el producto y su uso en entornos de atención médica diaria.

**Consideraciones Finales:**

el proceso de desarrollo participativo permitió la creación de una tecnología de asistencia adaptada a las necesidades de las enfermeras para la gestión y el seguimiento de los contactos en Atención Primaria.

## INTRODUCTION

The challenge of minimizing the epidemiological and social damage caused by leprosy is a priority among public policies focused on individual and collective health. It is a chronic disease that, although curable, remains endemic in several regions of the world, particularly in Brazil, where epidemiological and operational indicators are worrying^(1)^. It is associated with poverty and poor access to housing, food, healthcare and education, making it one of humanity’s oldest diseases^(2,3)^.

Caused by *Mycobacterium leprae*, it mainly affects the skin and peripheral nerves, and can also affect the upper respiratory tract mucosa, eyes, lymph nodes, testicles and internal organs, depending on a person’s level of immune resistance^(4)^. Leprosy presents with varying degrees of neuropathy, potentially causing changes in sensitivity in sensory, motor, and autonomic fibers, leading to severe physical disability and functional loss. It carries a significant stigma, being considered a neglected tropical disease because it affects the poorest segments of the population and receives little visibility^(5)^.

Considering such magnitude, leprosy was incorporated among the Sustainable Development Goals (SDGs) targets, which propose to eliminate neglected tropical diseases by the year 2030^(6)^. In support of the SDGs, the World Health Organization (WHO) established a leprosy elimination policy of diagnosing less than one case per 10,000 inhabitants^(7)^. This goal was followed by the Brazilian Ministry of Health, which formulated guidelines and strategies published in plans and ordinances with a time limit of achievement, currently, until the year 2030^(8)^. To this end, control actions were intensified with qualification of human resources for health education, incentives for research and implementation of normative instructions aimed at assistance^(8,9)^.

In line with the WHO’s postulate, the Ministry of Health created exclusive actions to eliminate leprosy at the national and local levels through the Brazilian National Strategy to Combat Leprosy, which aims to support managers, technicians and healthcare professionals in the preparation of state, regional and municipal plans, as well as the strategic actions to be developed^(9)^. Local peculiarities must be taken into account, with a view to organizing the service and qualifying the comprehensive care offered to people affected by leprosy and its complications within Primary Health Care (PHC) and specialized services^(10)^.

Among the strategic actions to achieve this goal, contact surveillance stands out. Its purpose is to identify new cases among those who live or have lived with a new case of leprosy for a long time and to implement preventive measures, such as prophylaxis with the Bacillus Calmette-Guérin (BCG) vaccine and dermatological and neurological examination. It also aims to identify possible sources of infection within the household (family) or outside the household (social), regardless of patients’ operational classification, whether paucibacillary or multibacillary^(11)^.

According to the Epidemiological Bulletin of Leprosy published by the Ministry of Health, since 2015, the state of Pará has shown a stationary trajectory in terms of the overall detection rate of new cases, remaining with a “high” indicator between 2016 and 2020. However, it is considered a fragile indicator when assessing stratification by territory, since the data show municipalities classified as hyperendemic and with a precarious proportion of contacts examined^(12)^.

In this context, among the control actions implemented in PHC, adequately addressing household and social contacts significantly contributes to the significant discovery of new cases, especially in contexts with relatively low or moderate endemicity. Contact management is a significant public health action, as it has the potential to generate counseling and systematic longitudinal monitoring of individuals and families at risk of illness. Therefore, strengthening these actions is important to accelerate disease control in line with the SDG agenda^(7,9,13)^.

In the context of PHC, the study observed nurses’ leading role in the Leprosy Control Program operationalization, developing management actions for planning, organizing, and assessing the service, as well as assistance activities, such as nursing consultation, investigation of suspected cases, with early recognition of signs and symptoms of leprosy, identification of signs of leprosy reactions, notification of confirmed cases, assessment and monitoring of peripheral nerve function, among others, contributing to the problem-solving nature of PHC, both individually and collectively^(13,14)^.

Thus, we understand the need for innovation in care practices utilizing health technologies that offer the possibility of achieving results based on everyday experience, enabling interventions in a given practical situation. Among these technologies, assistance ones stand out, which involve the construction of technical-scientific knowledge resulting from research, application of theories, and professionals’ and clients’ daily experience. Thus, assistive technology (AT) must enable interactional dimensions, allowing professionals to use their senses to choose and deliver care^(15)^.

It is important to emphasize that the use of AT for monitoring leprosy contacts is necessary given the role of nurses in contact surveillance and the improvement of disease control measures in the country. It is worth noting that the participatory construction of an AT in the creation process allows for interaction and knowledge exchange, as well as facilitating contact follow-up, contributing to disease control through qualified care^(16)^.

## OBJECTIVES

To construct, in a participatory manner, an AT to contribute to the nurses’ work process when managing leprosy contacts in PHC.

## METHODS

### Ethical care

The research was approved by the Research Ethics Committee of the undergraduate nursing program at the *Universidade do Estado do Pará*, and received institutional authorization from the Municipal Health Department of Bragança, Pará. Written informed consent was obtained from all study participants. To protect their identities, alphanumeric coding was adopted, using the letter “N” for nurse, followed by the sequential interview number.

### Study design

This is methodological research, with a participatory interface, developed in three sequential stages, namely: project presentation and individual data collection; technology construction according to what was proposed by participants; technology presentation to nurses for assessment, adjustments and obtaining the final product.

Due to this study’s design, it is important to note that it was not possible to use any guide from the EQUATOR network.

### Study setting, population and selection criteria

The study was conducted in an endemic municipality located in the Bragantina microregion of the Northeast mesoregion of the state of Pará. According to the state’s administrative regional division, it belongs to the 4^th^ Regional Health Center - Rio Caetés. Data collection took place in 19 Basic Health Units (BHUs), more precisely, with the Family Health teams (FHts) linked to these BHUs, accounting for 65.5% of the municipality’s 29 BHUs, nine in urban areas and ten in rural areas. The BHUs were randomly selected, considering the study’s operationalization within the pre-established data collection period, emphasizing the inclusion of nurses working with both urban and rural populations. It is important to clarify that the municipality has 100% coverage of the population with FHt.

Twenty-nine nurses participated, accounting for 46.7% of the total number of nurses working in the city’s FHts. The inclusion criterion was being a nurse working in urban and rural FHts with at least six months’ experience monitoring leprosy cases.

Sampling was performed for convenience, and to conclude data collection, data saturation was considered, i.e., when no new important element is found and the addition of new information is no longer necessary, as it does not alter the phenomenon studied and does not limit the scope of the objective^(17)^.

### Study protocol

In the first stage, the project was presented to the PHC coordinators at the Municipal Health Department to encourage them to inform and facilitate engagement with nurses working in FHts. Subsequently, the project was presented to nurses individually at their respective workplaces. Interviews were scheduled with those who agreed to participate, based on their availability, without disrupting their professional activities. Thus, all interviews were conducted at the health unit itself, at the end of the workday, in private rooms, ensuring participants’ comfort and privacy.

A semi-structured script developed by the authors was used, divided into two sections: the first to characterize participants and their academic and professional profile; and the second with open-ended questions to identify the concepts and practices regarding leprosy contact management, as well as the content and format to be observed when constructing AT. The interviews were conducted by the lead researcher and took place from March to October 2023, with an average duration of 13 minutes, and were recorded with consent.

In the second stage, the first version of the technology was developed, corresponding to a Contact Follow-up Book, created using Canva, a free online graphic design platform. It included content suggested by participants based on official Ministry of Health documents and an integrative literature review, which synthesized the scientific evidence to guide professional conduct in contact surveillance. The research question was constructed based on the adapted PICo strategy, considering P (person/problem), I (interest), and Co (context). In this case, P refers to nursing practice, I to leprosy contact surveillance, and Co to PHC. The question was posed: what practices should nurses develop for leprosy contact surveillance in PHC?

The databases selected were PubMed, Scientific Electronic Library Online (SciELO), and Latin American and Caribbean Literature in Health Sciences (LILACS). Articles in English, Portuguese, and Spanish, available in full, and published between 2018 and 2022, were included.

After this AT was developed based on the theoretical contexts described, the third stage involved qualifying the product with participants. This stage was held in December 2023. The AT was presented to nurses without selection criteria, as all participants were invited. Moreover, 55.1% (n=16) of the total participants in the first stage participated, although it was not possible to work with all of them due to scheduling constraints. The AT was assessed for contributions, synchronously, using Google Meet, by 11 participants, with another five contributing via email. Based on the participation percentage, it was understood that the group’s representativeness was ensured.

### Data analysis

The interviews were transcribed in full, reviewed to exclude language errors and spelling corrections, and constituted the *corpus* for processing in *Interface de R pour les Analyses Multidimensionnelles de Textes et de Questionnaires* (IRAMUTEQ) version 07 alpha 2.

Descending Hierarchical Classification (DHC) analysis was used. Based on the classes generated by DHC, the associations between significant words and text segments (TSs) in each class were interpreted to determine the meaning attributed to each. The classes were organized into thematic axes and discussed in light of relevant scientific literature and recommendations and guidelines of official documents on the topic.

## RESULTS

Female participants (79.32%; n=23), aged 25-35 years (55.17%; n=16), and professional experience in BHUs in the urban area (62.02%; n=18) predominated. Time since graduation in nursing ranged from one to 22 years, with a higher proportion of time over ten years (31.06%; n=9). The majority reported training at a specialization level (71.42%; n=21), with a small number in public health (17.24%; n=5). Job tenure in PHC ranged from six months to 22 years, with a predominance between one and two years (34.48%, n=10), and the length of experience with leprosy control actions, especially contact surveillance, was less than one year (37.98%; n=11).

**Figure 1:**
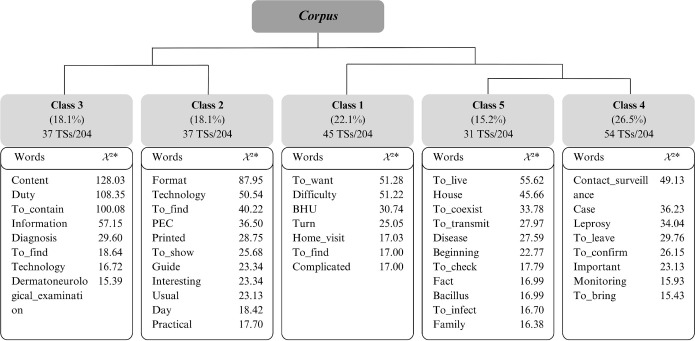
Descending Hierarchical Classification dendrogram, Belém, Pará, Brazil, 2023

The *corpus* was composed of 29 texts, divided into 255 TSs, of which 204 (80%) were used, giving rise to five classes by DHC (Figure 1). The *corpus* was divided into two *subcorpora:* the first formed by classes 1, 5 and 4, and the second, by classes 2 and 3.

*Chi-square test.

It should be noted that, to construct the thematic axes, the authors considered the classes in this order: 4, 5, 1, 2, and 3, due to the partition logic generated by processing in IRAMUTEQ. The thematic axes and their respective classes are presented below.

### Conceptions on leprosy contact surveillance and nurses’ decision-making

Composed of classes 4 and 5, it accounts for 41.7% of the total *corpus*, with 85 TSs. Together, the classes cover aspects related to nursing conduct and the concept of contact surveillance, highlighting the imminent risks to household contacts from nurses’ perspective. Actions developed for contact tracing, as well as the participation of other PHC professionals, stood out.

In class 4, the terms “contact_surveillance”, “case”, “leprosy”, “to_leave”, “to_confirm”, “important”, “monitoring”, and “to_bring” exhibited greater statistical significance, denoting the importance attributed by nurses to contact surveillance and that, from the diagnosis of a positive case, monitoring and attempts to link the contact to the BHU should begin, highlighting the role of community health workers (CHWs) in this process.

*The issue of leprosy contact surveillance is to track and actively search for these contacts; it is a way to monitor and discover new cases of the disease.* (N02)

*Here at the FHS* [Family Health Strategy]*, as soon as I have a leprosy case, I talk to that user to see how many contacts they have and explain the importance of these contacts coming to the unit. Then I contact the CHW to visit and explain to the user’s family that they should come to the unit. If that doesn’t work, I schedule a visit with the CHW, which is my last chance to reach these contacts.* (N20)

Nurses attribute an important role to the person undergoing leprosy treatment, recognizing their role as a link between the BHU and contacts, especially those within the home. They understand that it is the responsibility of the person undergoing treatment to guide the contact to the BHU, adopting a passive stance, first examining those who are taken to or even seek health services. It is observed that active search through home visits is neglected.

*When they don’t come, we actively seek out the patients with the CHWs. When the CHW can’t bring everyone, we have to make home visits and explain what leprosy is, the problems it can cause in their lives, and the harm it can cause to those around them.* (N05)

In class 5, representative words such as “to_live”, “house”, “to_coexist”, “to_transmit”, “disease”, and “family” refer to the concept of contact and the risks to which it is exposed. Although contact surveillance is considered an important strategy for controlling and eliminating leprosy as a public health problem, it appears that only intra-household contacts are valued over social contacts. This behavior reflects a management failure that can impact the effectiveness of control measures.

*From the moment a patient begins treatment, I explain this contact assessment to them, highlight its importance, and ask them to bring it to the BHU. I usually contact the CHW right away to check how many contacts actually live in the same house and whether there are children.* (N23)

### Operational difficulties and the work process in Primary Health Care

Formed by class 1, with 45 TSs, it deals with the operational obstacles of nurses’ work process in PHC, highlighting the difficulties in contact surveillance actions, as well as discussing the fragile relationship between leprosy contact and nurses.

TSs highlight the importance of assessing the vaccination status of household contacts, including the identification of BCG scars. Conversely, the recommendation to perform rapid testing for leprosy appears to be a hapax, meaning this strategy is not used in the management and triage of assessed contacts, reflecting a failure in contact surveillance and a lack of awareness among nurses.

*I check whether everyone is vaccinated with BCG and determine if a second dose is needed for prophylaxis. I also check how many contacts there are so I can request and administer the vaccine to those contacts.* (N11)

The words “difficulty”, “to_find”, and “complicated” refer to the difficulties in approaching contacts at the right time, with stigma surrounding leprosy recognized by participants as a factor hindering contact testing. Fear of being ill makes them uncooperative in undergoing testing, citing the fact that they do not recognize any signs or symptoms and, therefore, do not see the need to be tested. This is a challenge for the healthcare team, which must implement educational initiatives.

*I see that the biggest challenge is reaching out to contacts; making them understand the importance of this follow-up with them as well. I realize that they generally don’t want to come to the BHU due to prejudice against the disease and fear of being sick and not knowing it.* (N20)

In addition to the difficulties inherent in contact itself, poor working relationships are observed at the FHt, especially among physicians. Although nurses perceive the physician’s presence during home visits as positive, this action does not always materialize. Furthermore, it is recognized that staff turnover is a significant operational barrier, hindering the execution of mediumand long-term planning, in addition to compromising the bond with the population. This reality points to the FHt’s lack of character in terms of longitudinal care and multidisciplinary work, which leads to nurse overload and weakens leprosy contact surveillance actions.

*I also realize that staff turnover is a barrier because sometimes, out of the blue, we’re switched to another BHU, and information gets lost. We no longer know who our contacts are at this new BHU, and it becomes complicated; it’s as if everything is starting from scratch.* (N27)

*I also see a problem involving the rest of the FHS team in this matter of contacts. Sometimes, the doctor doesn’t want to make home visits with us or even assess contacts; this makes the process difficult.* (N29)

A relevant aspect recognized by nurses concerns the large geographic distances between BHUs and the homes of some index cases, a common characteristic of rural areas, especially in the Brazilian Amazon. Given the location of BHUs in rural and peri-urban areas, nurses face difficulties in conducting home visits, resulting in delays in assessing intra-household contacts due to the lack of or difficulty in obtaining transportation.

*Our assigned area has periurban characteristics: the houses are far from each other and even from BHU, and there are some farms and ranches. And because of the distance, it sometimes takes us a while to make a home visit.* (N23)

### Participatory construction of assistive technology for contact surveillance in Primary Health Care

Composed of classes 2 and 3, this axis addresses AT creation, with suggestions regarding the format they deemed appropriate for use in daily PHC and the most relevant topics to be addressed. From this perspective, these classes originated from questions related to AT usability based on daily PHC practices and the content and topics relevant to contact surveillance, as perceived by nurses.

In the lexical content of class 2, the words with the highest statistical significance were “technology”, “to_find”, “PEC”, “printed”, “to_show”, “guide”, “usual”, “day”, and “practical”. These words reflect issues related to the AT format. In class 3, the terms with the highest statistical significance were “content”, “duty”, “information”, “diagnosis”, “to_find”, “technology”, and “dermatoneurological_examination”, which reveal the content to be covered in the AT.

The words “PEC”, “printed”, “usual”, and “practical” refer to the AT format, reflecting the need for something practical for daily contact surveillance efforts. The most relevant aspect in deciding the tool’s format was the municipality’s reality, which concerns the difficulty of accessing mobile networks and the internet, compounded by intermittent power supply. Although all BHUs have internet access, it is not regular, especially in rural areas, where the largest number of them are located. This scenario was decisive in defining the AT format in printed format.

*I think it would be quite common to have something printed and that doesn’t require internet access, because sometimes we have problems with that here; sometimes the internet stops working and it’s difficult to even use the PEC* [*Prontuário Eletrônico do Cidadão*]*. So, I think it should be something printed.* (N20)

*I think this technology would be very practical; I think it would be possible to input something into the PEC* [*Prontuário Eletrônico do Cidadão*] *on a computer. But it would be interesting. The problem is that there are often power outages here, so perhaps something printed would be better.* (N25)

Concerning its content, it was considered pertinent to be a synthetic material that was easy to understand and visualize, covering aspects such as definition of diagnosis, immunoprophylaxis, rapid anti-*M. leprae* test, individual monitoring form, space for recording physical examination, dermatoneurological examination and frequency of monitoring.

*I think the individual profile of the contact should be addressed, including recent addresses and recent places of work, because this also poses a risk.* (N04)

*I think this technology should have content focused on dermato-neurological examination, as I said, and guidelines regarding this contact surveillance and follow-up over time.* (N24)

*In terms of content, I think it needs to have information about the disease, and space to register this contact, because the PEC* [*Prontuário Eletrônico do Cidadão*] *doesn’t have that. So, I think it would be interesting to have a little figure so we can signal the findings of the contact’s assessment. I think it would be good like that.* (N26)

In the product qualification stage, nurses were able to assess and suggest adjustments to content, information sequencing, message clarity, illustrations, and appearance, which were approved and considered clear and relevant to the context of its usability.

The final version was created, titled “*Caderno de Acompanhamento de Contatos de Hanseníase*” (Leprosy Contact Monitoring Book). The layout and formatting were designed in a vertical, A4 format, with informative texts about leprosy and the importance of contact surveillance and monitoring. The texts are in Garet font, ranging in size from 12 to 48, with 48 for titles and 12 for texts accompanying the illustrations.

The book includes a cover, back cover, presentation page, four instructional sections on leprosy contact surveillance, and specific monitoring forms for managing and following up contacts. Throughout the AT, there are QR codes with official Ministry of Health references for further information. The instructional sections cover case definition, contact classification, immunoprophylaxis, and rapid anti-*M. leprae* test applicability.

Additionally, the book includes contact follow-up forms with spaces for recording identifying data such as name, gender, date of birth, occupation, home address, work address, health unit, local CHW, name of index case, and date follow-up began. There is also space to indicate whether the contact is within the household or socially, as well as the rapid anti-*M. leprae* test result and whether BCG immunoprophylaxis is indicated.

The dermatological assessment record includes a specific space, with a graphic representation of the human body from the front and back, where clinical findings are easily indicated. The simplified neurological assessment follows Ministry of Health guidelines and must be performed and recorded for five years. To attract professionals’ attention, the “date” field is highlighted. There are also spaces for marking clinical findings, namely: leprosy confirmed; leprosy ruled out; inconclusive alterations. If lymphatic drainage microscopy is necessary, there is a specific space for recording the results as well as for nursing progress records.

## DISCUSSION

The participatory development of an AT for contact surveillance with PHC nurses was crucial to developing a product that was relevant to the local context and could be used in the daily routine of healthcare facilities. Evidencing the knowledge and practices related to monitoring leprosy contacts, understanding participants’ care process, proved crucial to its implementation.

The identification and monitoring of leprosy contacts are carried out in PHC, together with other actions recommended by the Brazilian National Leprosy Control Program^(18)^. The importance of contact surveillance actions developed in the region, as well as the participation of all team members in this process, is recognized for effective disease control. These concepts are in line with Ministry of Health guidelines and recommendations as a strategy to promote the effective decentralization of leprosy care to PHC, which should address approximately 80 to 90% of the population’s health needs^(11,18)^.

PHC also includes the key elements needed to improve health security and prevent health threats, as it is the gateway to the Brazilian Health System (In Portuguese, *Sistema Único de Saúde* - SUS) and is distributed throughout the region. It is essential to ensure professional qualification to adequately manage leprosy cases and to structure PHC services, incorporating the necessary technologies for timely and early diagnosis and treatment of the index case, in order to improve epidemiological indicators and improve contact surveillance actions^(13)^. In this way, PHC is configured as a point of attention in the fundamental assistance network for leprosy care^(19)^.

Considered a primary strategy for reducing leprosy burden, this study observed the presence of reactive actions regarding contact surveillance, a fact that does not contribute to effective measures for disease control in public health. Active contact tracing, when performed late and/or incorrectly, increases the chances of disease transmission within the family network, which can lead to the continuation of the chain of transmission between generations, exposing frequent social contacts to the risk of developing leprosy, as has been reported in the literature^(20-22)^.

The investigation of contacts of cases, considered one of the pillars of the general objective of the Brazilian National Strategy to Combat Leprosy, aims to strengthen early diagnosis through active search^(9)^. Thus, active search is a basic tool for tracking cases or contacts; however, a study highlights the difficulty nurses have in identifying possible injuries or complaints related to leprosy, which can influence diagnosis time, treatment initiation, and case worsening^(23)^. This finding demonstrates the need to promote professional qualification for the active search for new cases, which is one of the specific objectives of this pillar^(9)^.

Considering the public health problem at national and global levels, the strategy to reduce the burden of leprosy necessarily involves a qualified approach to contacts, given the probability of diagnosing cases, as early diagnosis contributes to reducing the chances of physical disabilities and impairments, having a favorable impact on the social, economic and psychological impact^(14,19)^. This strategy contributes to fulfilling the global elimination agenda, supporting the SDGs^(6)^.

Professional qualification involves strategic actions aimed at technical-scientific training, multisectoral and multiprofessional coordination regarding leprosy assessment and diagnosis procedures, as well as the integration of the approach with actions to combat stigma and discrimination existing in the historical context of leprosy, in addition to strengthening social inclusion^(9)^.

The Ministry of Health recognizes the need to establish contact investigation of leprosy cases in PHC, combined with best-practice approaches. To this end, it recommends developing documents that establish professionals’ responsibilities for active case finding and contact care, as well as guiding the approach to contacts regarding clinical assessment of signs and symptoms, health education, reception, bonding, and individual autonomy preservation^(9)^.

Among the actions carried out by nurses, it is noted that there is no standardization in the conduct of contact surveillance, distancing itself from that recommended by the Ministry of Health^(7,8)^. Operational failures in leprosy care and health surveillance are critical and likely account for the underestimation of endemic disease burden. This last perspective includes the coverage and quality of contact surveillance for effective disease control^(7,12,24)^.

A qualified dermatoneurological examination is essential for detecting leprosy, given that the diagnosis is mostly clinical^(10)^. However, only dermatological assessment was considered relevant during the physical examination. It is important to emphasize that diagnosis requires a thorough skin examination for spots, plaques, and nodules, as well as changes in thermal, pain, and/or tactile sensitivity. This is in addition to a thorough examination of the peripheral nerve trunks through palpation, as leprosy can present with skin changes and/or nerve trunk alterations^(9,21,24)^.

A thorough dermatoneurological assessment is also necessary to ensure the correct indication of immunoprophylaxis, through BCG vaccine and in accordance with vaccination history^(11)^. It is necessary to rule out the possibility of disease in order to proceed with immunoprophylaxis^(10)^. In nurses’ practice, the indication of immunoprophylaxis was observed as a priority, as in other studies^(24,25)^.

In the context of contact care, it is worth highlighting the rapid anti-*M. leprae* test, which is important for analyzing serology, since positive results indicate a greater risk of illness^(26,27)^. Brazil is a pioneer worldwide in incorporating a rapid test for detecting anti-*M. leprae* antibodies into the SUS as an auxiliary method for leprosy control actions^(28)^. This action should be used as a support tool in the assessment of contacts, in order to indicate the group to be actively monitored for the emergence of signs and symptoms of leprosy.

Nurses’ work process in FHTs encompasses leprosy care. Evidence suggests that nurses are capable of implementing the actions recommended by the Ministry of Health and are responsible for planning disease control actions for the community, users, and contacts of the index case^(10,11,13)^. However, work overload can hinder the execution of contact surveillance actions, mainly due to medical professionals’ low involvement in leprosy control actions, as evidenced in this study and in literature on the subject^(22,25)^.

The high turnover of professionals is also noteworthy, a factor that negatively contributes to the longitudinal monitoring of contacts, as evidenced in other studies^(13,14,22)^. It compromises the quality of control actions, as well as interferes with follow-up of contacts, also affecting continuing education, which becomes ineffective due to the excessive professional turnover^(14)^. In addition to the operational difficulties identified in healthcare services, social stigma contributes to the worsening of endemic disease in the country, making it necessary to guide the population with health education actions, and case diagnosis occasion should be valued^(25,29)^.

In the context of ruralization in the Brazilian Amazon, it is noted that the characteristics of territories, the places where they reside, sometimes geographically dispersed and distant from the BHU, making it difficult for both the population and the team to access homes, compromise contact tracing, which is aggravated by the lack of logistics that ensure the team’s movement^(10,12,14,20,22)^.

The implementation of new technologies that improve the early diagnosis of leprosy cases is recognized as an indispensable strategy for improving contact surveillance^(9)^. Thus, nurses’ leading role in contact tracing is notable, as it is understood that, by having an instrumental tool, they will be able to qualify their practice for this surveillance^(30)^.

The Contact Tracking Book, in turn, values participants’ knowledge in managing and tracking contacts, as it was developed based on their needs. Thus, AT supports the improvement of the work process, as recommended by the Ministry of Health, and, according to scientific literature, enhances nurses’ autonomy with a view to improving quality of care^(30,31)^.

Considering the Amazon territory singularities, the low supply of basic services, such as electricity and poor road infrastructure, makes up a reality that still impacts healthcare services in the countryside, hindering PHC computerization^(32)^. Added to this is the difficulty in planning and maintaining continuity of health actions^(22,32)^. Hence, the Ministry of Health recommends that states and municipalities develop their own strategies, based on the national strategy, according to the local epidemiological scenario, promoting reflections on tackling leprosy so that the planned actions are adapted to local needs^(9)^.

Therefore, the AT, developed participatively, aimed to combine professional experience with recommended contact surveillance measures, contributing to disease control and the implementation of longitudinal nursing care in PHC. The use of printed technologies is highlighted in the literature as beneficial for daily care activities, as well as a form of recordkeeping that facilitates communication between teams, and is relevant to the study context^(30)^.

### Study limitations

The fact that not 100% of nurses participated in the technology qualification process is considered a limitation. Nevertheless, it was found that those who participated provided sufficient results to support the participatory development process and qualify it satisfactorily.

### Contributions to nursing and health

The development of health technologies represents a significant advancement in nursing practice, particularly regarding ATs, which have the potential to standardize and serve as a source of communication within the multidisciplinary PHC team. It is noteworthy that their use in leprosy therapy can contribute, above all, to leprosy elimination agenda and the SDGs. The participatory interface, in turn, contributes to the effective daily use of the developed AT, which can be replicated in other settings and enable the improvement of leprosy control.

## FINAL CONSIDERATIONS

The creation of an AT in book form for better contact management demonstrates the potential for standardizing nursing practices regarding leprosy contact surveillance, given its participatory development. In this context, its usability as an AT is conceived as a tool for nursing work processes, aiming for safe and effective care, as well as facilitating communication and strengthening healthcare work, particularly in the care actions developed by PHC nurses. Investment in more research of this nature is highlighted, aiming to strengthen and improve leprosy control, especially contact monitoring, in PHC.

## Data Availability

The research data are available within the article.
