# Supplementary material for: Participatory construction of an assistive technology for leprosy contact surveillance
Source: Rev Bras Enferm. 2025 Dec 12;78(Suppl 4):e20240488. doi: 10.1590/0034-7167-2024-0488 (PMC12707850; doi:10.1590/0034-7167-2024-0488)
Supplement: Translation

# Translation: Construção participativa de uma tecnologia assistencial para vigilância de contatos de hanseníase

**DOI:** 10.1590/0034-7167-2024-0488pt

**Authors:** Breno Augusto Silva Duarte, Laura Maria Vidal Nogueira, Haroldo Gonçalves de Jesus, Ivaneide Leal Ataíde Rodrigues, Ingrid Bentes Lima, Raquel Gomes da Silva

**Affiliations:** I Universidade do Estado do Pará. Belém, Pará, Brasil

**Keywords:** Enfermagem, Atenção Primária à, Saúde, Busca de Comunicante, Hanseníase, Tecnologia em Saúde.

## Abstract

**Objetivos:**

construir, de forma participativa, uma tecnologia assistencial para contribuir com o processo de trabalho do enfermeiro diante do manejo de contatos de hanseníase na Atenção Primária à Saúde.

**Métodos:**

pesquisa metodológica, com interface participativa. Dados foram coletados entre março e outubro de 2023, em 19 Unidades Básicas de Saúde, com 29 enfermeiros, entrevistados individualmente. Utilizou-se o *software* IRAMUTEQ para análise de dados.

**Resultados:**

organizaram-se três eixos temáticos evidenciando: Concepções sobre vigilância de contatos de hanseníase e tomada de decisão; Dificuldades operacionais no processo de trabalho; e Desenvolvimento participativo da tecnologia assistencial. Os enfermeiros foram favoráveis à construção da tecnologia na forma de caderno de acompanhamento, apresentando sugestões de qualificação do produto para utilização no cotidiano dos serviços de saúde.

**Considerações Finais:**

a construção participativa possibilitou criar uma tecnologia assistencial condizente com as necessidades dos enfermeiros para manejo e monitoramento dos contatos na Atenção Primária à Saúde.

## INTRODUÇÃO

O desafio de minimizar danos epidemiológicos e sociais advindos da hanseníase é prioridade entre as políticas públicas voltadas à saúde individual e coletiva. Trata-se de doença de evolução crônica que, embora curável, permanece endêmica em várias regiões do mundo, essencialmente no Brasil, cujos indicadores epidemiológicos e operacionais são preocupantes^(1)^. Está associada à pobreza e ao acesso precário à moradia, alimentação, cuidados de saúde e educação, configurando-se como uma das doenças mais antigas da humanidade^(2,3)^.

Causada pelo *Mycobacterium leprae*, acomete, principalmente, a pele e os nervos periféricos, podendo atingir, também, a mucosa do trato respiratório superior, olhos, linfonodos, testículos e órgãos internos, conforme o grau de resistência imune da pessoa^(4)^. A doença cursa com neuropatia em graus variados, sendo capaz de provocar alterações de sensibilidade nas fibras sensitivas, motoras e autonômicas, gerando acentuada incapacidade física e perda funcional. Carrega consigo grande estigma, sendo considerada doença tropical negligenciada, por afetar as camadas mais pobres da população e receber pouca visibilidade^(5)^.

Considerando tal magnitude, a hanseníase foi incorporada entre as metas dos Objetivos de Desenvolvimento Sustentável (ODS), que propõem eliminar doenças tropicais negligenciadas até o ano de 2030^(6)^. Em prol dos ODS, a Organização Mundial da Saúde (OMS) estabeleceu como política de eliminação da hanseníase diagnosticar menos de um caso por 10 mil habitantes^(7)^. Essa meta foi seguida pelo Ministério da Saúde do Brasil, que formulou diretrizes e estratégias divulgadas em planos e portarias com limite temporal de alcance, atualmente, até o ano de 2030^(8)^. Para tanto, as ações de controle foram intensificadas com a qualificação de recursos humanos para educação em saúde, incentivo à pesquisa e implementação de instruções normativas voltadas à assistência^(8,9)^.

Em consonância com o postulado pela OMS, o Ministério da Saúde criou ações exclusivas para eliminação da doença no âmbito nacional e local por meio da Estratégia Nacional para Enfrentamento da Hanseníase, que objetiva subsidiar gestores, técnicos e profissionais de saúde quanto à elaboração dos planos estaduais, regionais e municipais, bem como das ações estratégicas a serem desenvolvidas^(9)^. Devem-se considerar as peculiaridades locais, tendo em vista a organização do serviço e qualificação da atenção integral ofertada à pessoa acometida pela doença e suas complicações no âmbito da Atenção Primária á Saúde (APS) e nos serviços especializados^(10)^.

No bojo das ações estratégicas para o alcance da meta, destaca-se a vigilância de contatos, cuja finalidade é a identificação de casos novos entre aqueles que convivem ou conviveram, de forma prolongada, com o caso novo de hanseníase, e implementar medidas preventivas, como a profilaxia com a vacina Bacilo de Calmette-Guérin (BCG) e o exame dermatoneurológico. Visa, também, descobrir possíveis fontes de infecção no domicílio (familiar) ou fora dele (social), independentemente da classificação operacional do doente, seja paucibacilar ou multibacilar^(11)^.

Segundo o Boletim Epidemiológico da Hanseníase divulgado pelo Ministério da Saúde, desde 2015, o estado do Pará apresenta uma trajetória estacionária no que se refere à taxa de detecção geral de casos novos, permanecendo com indicador “alto” entre 2016 e 2020. Entretanto, considera-se um indicador frágil quando se avalia a estratificação por território, visto que os dados mostram municípios classificados como hiperendêmicos e com precária proporção de contatos examinados^(12)^.

Nesse contexto, entre as ações de controle operacionalizadas na APS, a adequada abordagem de contatos domiciliares e sociais contribui sobremaneira com a descoberta significativa de casos novos, especialmente em contextos com endemicidade relativamente baixa ou moderada. A abordagem do contato é uma ação significativa para a saúde pública, na medida em que tem o potencial de gerar aconselhamento e seguimento longitudinal sistemático de pessoas e famílias expostas ao risco de adoecimento. Portanto, o fortalecimento dessas ações é importante para acelerar o controle da doença em consonância a agenda proposta pelos ODS^(7,9,13)^.

No contexto da APS, observa-se o protagonismo do enfermeiro na operacionalização do Programa de Controle da Hanseníase, desenvolvendo ações gerenciais de planejamento, organização e avaliação do serviço, bem como atividades assistenciais, como a consulta de enfermagem, investigação de casos suspeitos, com reconhecimento precoce dos sinais e sintomas da doença, identificação de sinais de reações hansênicas, notificação de casos confirmados, avaliação e monitoramento da função dos nervos periféricos, entre outras, contribuindo para o caráter resolutivo da APS, tanto individual quanto coletivamente^(13,14)^.

Dessa forma, entende-se a necessidade de inovação das práticas de cuidado ao utilizar tecnologias em saúde que oferecem a possibilidade de alcance de resultados a partir da experiência cotidiana, possibilitando provocar intervenções sobre uma determinada situação prática. Entre as tecnologias, destacam-se as assistenciais, que contemplam a construção de saber técnico-científico resultante de investigações, aplicações de teorias, e da experiência cotidiana dos profissionais e da clientela. Dessa forma, a tecnologia assistencial (TA) deve possibilitar dimensões interacionais, permitindo aos profissionais a utilização dos sentidos para a escolha e realização da assistência^(15)^.

Destaca-se que o uso de TA para o acompanhamento dos contatos de hanseníase se faz necessária tendo em vista o papel do enfermeiro frente a vigilância de contatos e o aprimoramento das ações para controle da doença no país. Vale ressaltar, que a construção participativa de uma TA no processo de criação, permite interação e troca de conhecimentos, assim como poderá facilitar o seguimento dos contatos, contribuindo para o controle da doença a partir da assistência qualificada^(16)^.

## OBJETIVOS

Construir, de forma participativa, uma TA para contribuir com o processo de trabalho do enfermeiro diante do manejo de contatos de hanseníase na Atenção Primária à Saúde.

## MÉTODOS

### Cuidados éticos

A pesquisa foi aprovada pelo Comitê de Ética em Pesquisa do curso de graduação em enfermagem da Universidade do Estado do Pará, e obteve autorização institucional da Secretaria Municipal de Saúde de Bragança, Pará. O Termo de Consentimento Livre e Esclarecido foi obtido por escrito por todos os participantes do estudo e, para resguardar suas identidades, foi adotado o método de codificação alfanumérico, utilizando a letra “E”, de enfermeiro, seguido pelo número sequencial das entrevistas.

### Desenho do estudo

Esta é uma pesquisa metodológica, com interface participativa, desenvolvida em três etapas sequenciais, a saber: apresentação do projeto e coleta de dados individual; construção da tecnologia, de acordo com o proposto pelos participantes; apresentação da tecnologia aos enfermeiros para apreciação, ajustes e obtenção do produto final.

Em razão do tipo de estudo, destaca-se que não foi possível utilizar nenhum guia da rede EQUATOR.

### Cenário do estudo, população e critérios de seleção

A pesquisa foi realizada em município endêmico, localizado na mesorregião Nordeste do estado do Pará, microrregião Bragantina, e conforme a divisão regional administrativa do estado, pertence ao 4º Centro Regional de Saúde - Rio Caetés. A coleta de dados se deu em 19 Unidades Básicas de Saúde (UBS), mais precisamente, junto às equipes de Saúde da Família (eSF) vinculadas a essas UBS, representando 65,5% do total de 29 UBS do município, sendo nove em zona urbana e dez em zona rural. A escolha das UBS se deu de forma aleatória, considerando a operacionalização do estudo no período pré-estabelecido para coleta dos dados, valorizando a inclusão de enfermeiros atuando junto à população urbana e rural. Faz-se necessário esclarecer que o município conta com cobertura de 100% da população com eSF.

Participaram 29 enfermeiros, correspondendo a 46,7% do total que atuam nas eSFs do município. Como critério de inclusão, considerou-se ser enfermeiro atuante na eSF urbana e rural com experiência no acompanhamento de casos de hanseníase há, pelo menos, seis meses.

A amostragem ocorreu por conveniência e, para encerrar a coleta de dados, considerou-se a saturação dos dados, ou seja, quando nenhum novo elemento importante é encontrado e o acréscimo de novas informações deixa de ser necessário, pois não altera o fenômeno estudado e não limita o alcance do objetivo^(17)^.

### Protocolo do estudo

Na primeira etapa, o projeto foi apresentado à coordenação da APS na Secretaria Municipal de Saúde, a fim de sensibilizá-los a informar e viabilizar a aproximação com os enfermeiros atuantes nas eSFs. Posteriormente, o projeto foi apresentado aos enfermeiros, em seus respectivos locais de trabalho, de forma individual, e com os que se disponibilizaram a participar, foram agendadas as entrevistas conforme disponibilidade, sem que houvesse prejuízos nas atividades profissionais. Assim, todas as entrevistas foram realizadas na própria unidade de saúde, ao final do expediente, em salas reservadas, assegurando conforto e privacidade ao participante.

Utilizou-se roteiro semiestruturado elaborado pelos autores, dividido em dois blocos: o primeiro para caracterização dos participantes e perfil acadêmico-profissional; e o segundo com perguntas abertas para identificar as concepções e práticas diante do manejo dos contatos de hanseníase, assim como conteúdo e formato a serem observados para construção da TA. As entrevistas foram conduzidas pelo pesquisador responsável e ocorreram no período de março a outubro de 2023, com duração média de 13 minutos, gravadas mediante consentimento.

Na segunda etapa, construiu-se a primeira versão da tecnologia, correspondente a um Caderno de Acompanhamento dos Contatos, elaborada por meio do Canva, plataforma *online* e gratuita de *design* gráfico, incluindo conteúdos sugeridos pelos participantes com base em documentos oficiais do Ministério da Saúde e revisão integrativa da literatura, que sintetizou as evidências científicas para orientar a conduta profissional na vigilância de contatos. Construiu-se a questão de pesquisa baseada na estratégia PICo adaptada, considerando P (pessoa/ problema), I (interesse) e Co (contexto). Neste caso, P refere-se à prática de enfermeiros, I, à vigilância de contatos de hanseníase, e Co, à APS. Estabeleceu-se a pergunta: quais práticas devem ser desenvolvidas por enfermeiros para vigilância de contatos de hanseníase na APS?

Elegeram-se as bases de dados PubMed, *Scientific Eletronic Library Online* (SciELO) e Literatura Latino-Americana e do Caribe em Ciências da Saúde (LILACS). Incluíram-se artigos nos idiomas inglês, português e espanhol, disponíveis na íntegra e publicados no período de 2018 a 2022.

Após a construção da TA, fundamentada nos contextos teóricos descritos, ocorreu a terceira etapa, que diz respeito à qualificação do produto com os participantes, realizada em dezembro de 2023, ocasião em que foi apresentada aos enfermeiros sem critérios de seleção, visto que todos foram convidados, havendo adesão de 55,1% (n=16) do total de participantes da primeira etapa, sendo que não foi possível trabalhar com 100% deles mediante impossibilidades de agenda. A TA foi apreciada para contribuições, de forma síncrona, com os recursos da plataforma *Google Meet*, por 11 participantes, com outros cinco contribuindo via e-mail. Mediante o percentual de participação, entendeu-se que foi assegurada a representatividade do grupo.

### Análise de dados

As entrevistas foram transcritas integralmente, revisadas para exclusão de vícios de linguagem e correções ortográficas, e constituíram o *corpus* para processamento no *software Interface de R pour les Analyses Multidimensionnelles de Textes et de Questionnaires* (IRAMUTEQ), versão 07 alpha 2.

Utilizou-se a análise por Classificação Hierárquica Descendente (CHD). A partir das classes geradas pela CHD, foram interpretadas as associações entre as palavras significativas e segmentos de texto (STs) de cada classe, com o intuito de verificar o significado atribuído a cada uma delas. As classes foram organizadas em eixos temáticos e discutidas à luz da literatura científica pertinente e das recomendações e orientações de documentos oficiais sobre o tema.

## RESULTADOS

Entre os participantes, predominou o sexo feminino (79,32%; n=23), na faixa etária 25-35 anos (55,17%; n=16) e atuação profissional em UBS na zona urbana (62,02%; n=18). O tempo de graduação em enfermagem, variou entre um e 22 anos, com maior proporção para tempo superior a dez anos (31,06%; n=9). A maioria referiu formação em nível de especialização (71,42%; n=21), sendo um pequeno número em saúde coletiva (17,24%; n=5). O tempo de atuação na APS variou de seis meses a 22 anos, com predominância entre um e dois anos (34,48%; n=10), e o tempo de experiência com as ações de controle da hanseníase, sobretudo, a vigilância de contatos, foi inferior a um ano (37,98%, n=11).

O *corpus* foi composto por 29 textos, desdobrados em 255 STs, das quais 204 (80%) foram aproveitadas, dando origem a cinco classes pela CHD (Figura 1). O *corpus* foi dividido em dois *subcorpora:* o primeiro formado pelas classes 1, 5 e 4, e o segundo, pelas classes 2 e 3.

**Figura 1:** Dendrograma da Classificação Hierárquica Descendente, Belém, Pará, Brasil, 2023

*Teste qui-quadrado.

Ressalta-se que, para construção dos eixos temáticos, os autores consideraram as classes nesta ordem: 4, 5, 1, 2 e 3, em virtude da lógica de partição gerada pelo processamento no IRAMUTEQ. A seguir, apresentam-se os eixos temáticos com suas respectivas classes.

### Concepções sobre vigilância de contatos de hanseníase e tomada de decisão do enfermeiro

Formado pelas classes 4 e 5, representa 41,7% do total do *corpus*, com 85 STs. Juntas, as classes contemplam aspectos relacionados à conduta dos enfermeiros e à concepção sobre a vigilância de contatos, destacando os riscos iminentes para os contatos intradomiciliares sob a ótica do enfermeiro. Emergiram, também, as ações desenvolvidas para rastreamento de contatos, bem como a participação de outros profissionais da APS.

Na classe 4, os termos “vigilância_de_contatos”, “caso”, “hanseníase”, “partir”, “confirmar”, “importante”, “monitoramento” e “trazer” exibiram maior significância estatística, denotando a importância atribuída pelos enfermeiros sobre a vigilância de contatos e que, a partir do diagnóstico de um caso positivo, deve-se iniciar o monitoramento e a tentativa de vinculação do contato à UBS, sendo destacado o papel do agente comunitário de saúde (ACS) nesse processo.

*A questão da vigilância de contatos de hanseníase é fazer esse rastreamento e busca ativa desses contatos, é uma forma de monitorar e descobrir casos novos da doença.* (E02)

*Aqui na ESF* [Estratégia Saúde da Família], *assim que eu tenho um caso de hanseníase, eu converso com esse usuário pra ver quantos são esses contatos e falo sobre a importância de esses contatos para se consultarem na unidade. Aí eu aciono o ACS pra fazer visita e explicar para a família do usuário vir até a unidade. Se não der certo, eu agendo uma visita com o ACS, que é a minha última oportunidade de conseguir chegar até esses contatos.* (E20)

Os enfermeiros atribuem à pessoa em tratamento para hanseníase papel importante, reconhecendo ser um elo entre a UBS e os contatos, principalmente os intradomiciliares. Entendem ser atribuição da pessoa em tratamento conduzir o contato até a UBS, colocando-se em conduta de passividade, examinando, *a priori*, aqueles que são levados ou mesmo procuram o serviço de saúde. Observa-se que a busca ativa através da visita domiciliar é deixada em segundo plano.

*Quando eles não vêm, aí a gente já faz essa busca ativa com os ACS. Quando o ACS não consegue trazer todos, nós que temos que ir na visita domiciliar e já explica o que é a hanseníase, a problemática que ela pode trazer para a sua vida e o prejuízo a quem está ao redor.* (E05)

Na classe 5, palavras representativas como “morar”, “casa”, “conviver”, “transmitir”, “doença” e “família” remetem ao conhecimento do conceito de contato e dos riscos aos quais está exposto. Embora a vigilância de contatos seja considerada estratégia importante para o controle e eliminação da hanseníase como problema de saúde pública, constata-se que são valorizados apenas os contatos intradomiciliares em detrimento dos sociais. Tal conduta remonta à falha de manejo que pode impactar a eficácia das ações de controle.

*A partir do momento que esse usuário inicia o tratamento, eu explico sobre essa avaliação dos contatos para ele, falo da importância e peço para que ele traga até a UBS. Geralmente, eu aciono logo o ACS também para já verificar quantos contatos moram de fato na mesma casa, se tem criança.* (E23)

### Dificuldades operacionais e o processo de trabalho na Atenção Primária à Saúde

Formada pela classe 1, com 45 STs, trata sobre os entraves operacionais do processo de trabalho do enfermeiro na APS, destacando as dificuldades nas ações de vigilância dos contatos, bem como discutindo a frágil relação entre o contato de hanseníase e o enfermeiro.

Nos STs, é destacada a valorização da situação vacinal dos contatos intradomiciliares, buscando a cicatriz da vacina BCG. Em contrapartida, a recomendação para realização do teste rápido para hanseníase surge como *hápax*, ou seja, essa estratégia não é utilizada no manejo e triagem dos contatos avaliados, remetendo a uma falha na vigilância dos contatos e ao desconhecimento desta pelos enfermeiros.

*Eu investigo se todos estão vacinados com BCG e vejo também a necessidade de refazer a dose como profilaxia. Também verifico quantos contatos são para que eu posso solicitar e fazer a vacina nesses contatos.* (E11)

As palavras “dificuldade”, “encontrar” e “complicado” remetem às dificuldades para abordagem dos contatos em momento oportuno, sendo o estigma que envolve a hanseníase reconhecido pelos participantes como fator dificultador para o exame dos contatos. O medo de estar doente torna-os pouco colaborativos na realização do exame, sob justificativa que não reconhecem nenhum sinal ou sintoma e que, por isso, não veem necessidade de serem examinados. Trata-se de um desafio para a equipe de saúde, que deve implementar ações educativas.

*Eu vejo que a dificuldade maior é chegar até os contatos; é fazê-los entender a importância desse acompanhamento com eles também. Eu percebo que geralmente eles não querem vir na UBS devido ao preconceito com a doença e por medo de estarem doentes e não saberem.* (E20)

Para além das dificuldades inerentes ao próprio contato, observam-se relações de trabalho pouco comprometidas na eSF, principalmente do profissional médico. Embora os enfermeiros percebam como positiva a presença do médico durante a visita domiciliar, essa ação nem sempre se concretiza. Também, é reconhecido que a rotatividade dos profissionais é uma barreira operacional de grande relevância, dificultando a execução de planejamento a médio e longo prazo, além de comprometer o vínculo com a população. Essa realidade remete à descaracterização da eSF sob a luz da longitudinalidade do cuidado e atuação multiprofissional, que induz à sobrecarga do enfermeiro e fragiliza as ações da vigilância de contatos de hanseníase.

*Eu percebo também que a rotatividade de profissionais é uma barreira porque, às vezes, do nada, trocam a gente de UBS e as informações se perdem. A gente não sabe mais quem são os contatos nessa nova UBS e fica complicado; é como se tudo começasse do zero.* (E27)

*Também vejo como problema envolver os demais da equipe da ESF nessa questão dos contatos. Por vezes, o médico não quer ir fazer visita domiciliar junto com a gente ou sequer avaliar os contatos; isso torna o processo dificultoso.* (E29)

Um aspecto relevante, reconhecido pelos enfermeiros, diz respeito às grandes distâncias geográficas entre a UBS e o domicílio de alguns casos índices, uma característica comum de territórios ruralizados, sobretudo na Amazônia brasileira. Considerando a localização das UBS em áreas rurais e periurbanas, os enfermeiros enfrentam dificuldades para realização da visita domiciliar, acarretando demora na avaliação dos contatos intradomiciliares devido à falta ou dificuldade para conseguir transporte.

*A nossa área adscrita tem características periurbanas: as casas são distantes uma da outra e até mesmo da UBS, tem uns sítios e fazendas. E devido à distância, às vezes a gente demora a conseguir fazer a visita domiciliar.* (E23)

### Construção participativa da tecnologia assistencial para vigilância de contatos na Atenção Primária à Saúde

Formado pelas classes 2 e 3, este eixo trata da criação da TA, com as sugestões referentes ao formato que julgaram adequado para uso no cotidiano da APS e aos temas mais relevantes a serem contemplados. Nessa perspectiva, essas classes se originaram dos questionamentos relacionados à usabilidade da TA com base no cotidiano da APS, aos conteúdos e temas pertinentes à vigilância de contatos, conforme a visão dos enfermeiros.

No conteúdo lexical da classe 2, observa-se que as palavras com maior relevância estatística foram “tecnologia”, “achar”, “PEC”, “impresso”, “mostrar”, “guia”, “usual”, “dia” e “prático”. Estas palavras refletem questões referentes ao formato da TA. Na classe 3, os termos com maior relevância estatística foram “conteúdo”, “dever”, “informação”, “diagnóstico”, “achar”, “tecnologia” e “exame dermatoneurológico”, que revelam o conteúdo a ser contemplado na TA.

As palavras “PEC”, “impresso”, “usual” e “prático” remetem ao formato da TA, entendendo-se a necessidade de algo prático para o dia a dia nas ações de vigilância dos contatos. O aspecto mais relevante para a decisão do formato da ferramenta foi a realidade do município, que diz respeito à dificuldade de acesso à rede móvel e à internet, potencializado pelo fornecimento de energia elétrica de forma intermitente. Embora todas as UBS contem com acesso à internet, não é regular, sobretudo na zona rural, onde está localizado o maior número delas. Tal cenário foi decisivo para definição do tipo-forma da TA no formato impresso.

*Eu acho que seria bem usual algo impresso e que não precise de internet, porque às vezes a gente encontra problema com isso aqui; às vezes, a internet para de funcionar e fica complicado até utilizar o PEC* [Prontuário Eletrônico do Cidadão]. *Então, acho que deve ser algo impresso.* (E20)

*Acho que essa tecnologia deve ser algo bem prático; acho que no computador mesmo, não sei se dá pra inserir algo no PEC* [Prontuário Eletrônico do Cidadão]. *Mas seria interessante, o entrave é que aqui costuma faltar energia, então talvez algo impresso seja melhor.* (E25)

Em relação ao seu conteúdo, considerou-se pertinente ser um material sintético de fácil compreensão e visualização, contemplando aspectos como definição do diagnóstico, imunoprofilaxia, teste rápido anti-*M. leprae*, ficha de acompanhamento individual, espaço para registro do exame físico, exame dermatoneurológico e periodicidade do acompanhamento.

*Acho que deveriam ser abordados o perfil individual do contato, levantamento dos últimos endereços, locais de trabalho recentes, porque isso oferece um risco também*. (E04)

*Acho que essa tecnologia deve ter conteúdo voltados ao exame dermatoneurológico, como eu falei, e orientações em relação a essa vigilância de contato e o seguimento ao longo do tempo.* (E24)

*De conteúdo, eu vejo que precisa ter informações sobre a doença, espaço para cadastrar esse contato, porque, no PEC* [Prontuário Eletrônico do Cidadão]*, não tem isso, então acho que seria interessante um bonequinho também pra gente ir sinalizando os achados da avaliação do contato. Acho que ficaria bom assim.* (E26)

Na etapa de qualificação do produto, os enfermeiros puderam apreciar e sugerir adequações de conteúdo, sequenciamento de informações, clareza da mensagem, ilustrações e aparência, sendo aprovada e considerada clara e pertinente para o contexto de sua usabilidade.

Construiu-se a versão final, que foi intitulada “Caderno de Acompanhamento de Contatos de Hanseníase”. O *layout* e diagramação foram concebidos no formato vertical, tamanho A4, com textos informativos sobre hanseníase e a importância da vigilância de contatos e monitoramento. Os textos estão em fonte *Garet*, variando os tamanhos entre 12 e 48, sendo 48 para títulos, e 12, para textos que acompanham as ilustrações.

O caderno conta com capa, contracapa, página de apresentação, quatro seções instrutivas sobre a vigilância de contatos de hanseníase e fichas de acompanhamento próprias para o manejo e seguimento dos contatos. Ao longo da TA, há *QR codes* com referências oficiais do Ministério da Saúde para aprofundamento dos conteúdos. As seções instrutivas contemplam definição de caso, classificação dos contatos, imunoprofilaxia e aplicabilidade do teste rápido anti-*M. leprae.*

Em adição, o caderno contempla as fichas de acompanhamento dos contatos com espaços para registro de dados de identificação como nome, gênero, data de nascimento, ocupação, endereço do domicílio, endereço profissional, unidade de saúde de vinculação, ACS da área, nome do caso índice e data de início do acompanhamento. Consta, ainda, espaço para assinalar se é contato intradomiciliar ou social, além do registro do resultado do teste rápido anti-*M. leprae* e se há indicação de imunoprofilaxia com a vacina BCG.

O registro da avaliação dermatológica conta com espaço específico, em representação gráfica do corpo humano de frente e de costas, onde serão sinalizados de forma facilitada os achados clínicos. A avaliação neurológica simplificada seguirá o roteiro preconizado pelo Ministério da Saúde, devendo ser realizada e registrada durante cinco anos, e, para chamar a atenção do profissional, o campo “data” encontra-se em destaque. Há também espaços para marcação de conclusões clínicas, a saber: hanseníase confirmada; hanseníase descartada; alterações inconclusivas. Caso se faça necessário realizar a baciloscopia de linfa, há campo específico para anotação dos resultados, assim como para os registros da evolução da enfermagem.

## DISCUSSÃO

A construção participativa de uma TA para vigilância de contatos com os enfermeiros da APS foi fundamental para se pensar em um produto condizente com a realidade local e que pode ser utilizado no cotidiano das unidades de saúde. Evidenciar os saberes e práticas relacionadas ao monitoramento dos contatos de hanseníase, compreendendo o processo de cuidar pelos participantes, mostrou-se importante para sua realização.

A identificação e monitoramento dos contatos de hanseníase são realizados na APS, juntamente com outras ações preconizadas pelo Programa Nacional de Controle da Hanseníase^(18)^. É reconhecida a importância das ações de vigilância de contatos desenvolvidas no território, bem como da participação de todos os profissionais da equipe neste processo para o efetivo controle da doença. Tais concepções vão ao encontro das orientações e recomendações preconizadas pelo Ministério da Saúde como estratégia para promoção da efetiva descentralização do cuidado em hanseníase para a APS, devendo responder por cerca de 80 a 90% das necessidades de saúde da população^(11,18)^.

A APS também inclui os principais elementos necessários para melhorar a segurança sanitária e prevenir ameaças à saúde, por ser porta de entrada do Sistema Único de Saúde (SUS) e capilarizada no território. É imprescindível garantir a qualificação dos profissionais de saúde para manejar adequadamente os casos de hanseníase e a estruturação dos serviços da APS, incorporando as tecnologias necessárias para estabelecer o diagnóstico e o tratamento de maneira oportuna e precoce ao caso índice, a fim de melhorar os indicadores epidemiológicos, bem como qualificar as ações voltadas a vigilância de contatos^(13)^. Dessa forma, a APS se configura como ponto de atenção da rede assistencial fundamental para o cuidado em hanseníase^(19)^.

Tida como estratégia primordial para redução da carga de hanseníase, neste estudo, observa-se a presença de ações reativas no que tange à vigilância de contatos, fato este que não contribui com medidas efetivas para o controle da doença na saúde pública. A busca ativa, quando realizada de forma tardia e/ou incorreta, aumenta as chances de transmissão da doença na rede familiar, o que pode acarretar manutenção da cadeia de transmissão entre as gerações, expondo os contatos sociais frequentes ao risco de desenvolver a doença, conforme vem sendo relatado na literatura^(20-22)^.

A investigação de contatos dos casos, considerada um dos pilares do objetivo geral da Estratégia Nacional para Enfrentamento da Hanseníase, visa ao fortalecimento do diagnóstico precoce mediante a busca ativa^(9)^. Assim, a busca ativa é uma ferramenta básica para rastreio de casos ou contatos; no entanto, estudo pontua a dificuldade dos enfermeiros na identificação de possíveis lesões ou queixas relacionadas à hanseníase, o que pode influenciar o tempo para diagnóstico, início de tratamento e agravamento do caso^(23)^. Esse achado demostra a necessidade de promover a qualificação de profissionais para busca ativa de casos novos, sendo um dos objetivos específicos desse pilar^(9)^.

Considerando o problema de saúde pública em nível nacional e global, a estratégia para redução da carga da hanseníase passa, necessariamente, pela abordagem qualificada dos contatos, visto a probabilidade de diagnosticar casos, já que o diagnóstico precoce contribui para redução das chances de incapacidades físicas e deficiências, repercutindo favoravelmente no impacto social, econômico e psicológico^(14,19)^. Tal estratégia contribui para o cumprimento da agenda de eliminação mundial, corroborando os ODS^(6)^.

A qualificação dos profissionais envolve ações estratégicas voltadas à capacitação técnico-científica, à articulação multissetorial e multiprofissional quanto aos procedimentos de avaliação e diagnóstico da hanseníase, bem como à integração da abordagem com ações de combate ao estigma e à discriminação existentes no contexto histórico da hanseníase, além do fortalecimento da inclusão social^(9)^.

O Ministério da Saúde reconhece a necessidade de estabelecer a investigação de contatos de caso de hanseníase na APS aliada às abordagens baseadas em boas práticas. Para isso, pontua como estratégia a elaboração de documentos que estabeleçam as atribuições dos profissionais para busca ativa de casos e de assistência dos contatos, além de nortear a abordagem do contato quanto à avaliação clínica de sinais e sintomas, educação em saúde, acolhimento, vínculo, e preservação da autonomia do indivíduo^(9)^.

Entre as ações realizadas pelos enfermeiros, nota-se que não há padronização na conduta de vigilância dos contatos, distanciando-se do preconizado pelo Ministério da Saúde^(7,8)^. Falhas operacionais de atenção e vigilância à saúde voltada para hanseníase são críticas e provavelmente respondem pelo subdimensionamento da carga da endemia. Inserem-se, nesta última perspectiva, a cobertura e a qualidade da vigilância de contatos para o efetivo controle da doença^(7,12,24)^.

O exame dermatoneurológico qualificado é essencial para detecção da hanseníase, tendo em vista que o diagnóstico é majoritariamente clínico^(10)^. Entretanto, observou-se valorização somente da avaliação dermatológica por ocasião do exame físico. É válido enfatizar que, para o diagnóstico, é necessário realizar exame minucioso da pele em busca de manchas, placas, nódulos, bem como alterações de sensibilidade térmica, dolorosa e/ou tátil, além do exame minucioso dos troncos nervosos periféricos por meio da palpação, pois a hanseníase pode se apresentar como alterações na pele e/ou com alteração de tronco nervoso^(9,21,24)^.

A realização da avaliação dermatoneurológica de forma criteriosa é necessária também para que se faça a indicação correta da imunoprofilaxia, através da vacina BCG e de acordo com o histórico vacinal^(11)^. Faz-se necessário descartar a possibilidade de doença para proceder à imunoprofilaxia^(10)^. Observou-se na prática dos enfermeiros a indicação da imunoprofilaxia, prioritariamente, assim como em outros estudos^(24,25)^.

Merece destaque, no contexto da atenção aos contatos, a realização do teste rápido anti-*M. leprae*, importante para analisar a sorologia, visto que resultados positivos exibem maior risco de adoecimento^(26,27)^. O Brasil é pioneiro, mundialmente, por incorporar no âmbito do SUS um teste rápido para detecção de anticorpos anti-*M. leprae* como método auxiliar as ações de controle da hanseníase^(28)^. Esta ação deve ser utilizada como ferramenta de apoio na avaliação de contatos, a fim de indicar o grupo a ser monitorado ativamente quanto ao surgimento de sinais e sintomas da doença.

Na eSF, o processo de trabalho do enfermeiro perpassa o cuidado em hanseníase. As evidências afirmam que o enfermeiro tem capacidade para desenvolver as ações indicadas pelo Ministério da Saúde, e é responsável por planejar as ações para controle da doença para a comunidade, para os usuários e aos contatos do caso índice^(10,11,13)^. Contudo, a sobrecarga de trabalho pode prejudicar a execução das ações de vigilância de contatos, principalmente pelo baixo envolvimento dos profissionais médicos nas ações de controle da hanseníase, conforme evidenciado neste estudo e em literatura sobre o tema^(22,25)^.

Destaca-se ainda a alta rotatividade de profissionais, um fator que contribui negativamente para o acompanhamento longitudinal dos contatos, como evidenciado em outros estudos^(13,14,22)^. Compromete a qualidade das ações de controle, bem como interfere no seguimento dos contatos, afetando também a educação permanente, que se torna ineficaz pela excessiva troca de profissionais^(14)^. Para além das dificuldades operacionais identificadas nos serviços de saúde, o estigma social contribui ao agravamento da endemia no país, tornando-se necessário orientar a população com ações de educação em saúde, devendo ser valorizada a ocasião do diagnóstico do caso^(25,29)^.

No contexto da ruralização na Amazônia brasileira, nota-se que as características dos territórios, os locais onde residem, algumas vezes dispersos geograficamente, e distantes das UBS, dificultando o acesso tanto da população quanto da equipe ao domicílio, comprometem o rastreamento dos contatos, sendo agravados pela inexistência de logística que assegure o deslocamento da equipe^(10,12,14,20,22)^.

A implementação de novas tecnologias que aprimorem o diagnóstico precoce dos casos de hanseníase é reconhecida como uma estratégia indispensável para o aprimoramento da vigilância de contatos^(9)^. Dessa forma, o protagonismo do enfermeiro no rastreamento de contatos é notório, pois entende-se que, ao dispor de uma ferramenta instrumentalizadora, poderá qualificar sua prática para essa vigilância^(30)^.

O Caderno de Acompanhamento dos Contatos, por sua vez, valoriza os saberes dos participantes no manejo e seguimento dos contatos, já que foi construído a partir das suas necessidades. Dessa forma, a TA se apoia no aperfeiçoamento do processo de trabalho, como preconizado pelo Ministério da Saúde, e potencializa, conforme a literatura científica, a autonomia do enfermeiro com vistas à qualidade da assistência^(30,31)^.

Considerando as singularidades do território Amazônico, a baixa oferta de serviços básicos, a exemplo de energia elétrica e pouca infraestrutura nas estradas, compõe uma realidade que ainda impacta os serviços de saúde na interiorização, dificultando a informatização da APS^(32)^. Soma-se a isso a dificuldade de planejamento e continuidade das ações de saúde^(22,32)^. Nesse sentido, o Ministério da Saúde recomenda que estados e municípios elaborem suas próprias estratégias, com base na estratégia nacional, segundo o cenário epidemiológico local, promovendo reflexões sobre o enfrentamento da hanseníase, de forma que as ações planejadas sejam adaptadas às necessidades locais^(9)^.

Portanto, a TA, construída participativamente, visou aliar a experiência profissional com medidas preconizadas para vigilância dos contatos, contribuindo para o controle da doença e efetivação do cuidado longitudinal do enfermeiro na APS. O uso de tecnologias em formato impresso é ressaltado na literatura como benéfico para as atividades assistenciais diárias, bem como uma forma de registro que facilita a comunicação entre as equipes, sendo pertinente ao contexto do estudo^(30)^.

### Limitações do estudo

Entende-se como limitação o fato da não participação de 100% dos enfermeiros na qualificação da tecnologia. Ainda assim, constatou-se que os que participaram propiciaram resultados suficientes para embasar o processo de construção participativa e qualificá-la a contento.

### Contribuições para as áreas da enfermagem e saúde

A construção de tecnologias em saúde figura como um avanço relevante na prática profissional do enfermeiro, principalmente no que tange às tecnologias assistenciais, que têm potencial para padronizar e servir como fonte de comunicação entre a equipe multidisciplinar da APS. Destaca-se que seu uso voltado para a hanseníase pode contribuir, sobretudo, para a agenda de eliminação da doença em prol dos ODS. A interface participativa, por sua vez, contribui para a efetivação do uso cotidiano da TA desenvolvida, podendo ser replicada para outras realidades e possibilitando qualificar o controle da hanseníase.

## CONSIDERAÇÕES FINAIS

A criação da TA em forma de caderno para melhor controle dos contatos demonstra potencial para padronizar as condutas de enfermagem em relação à vigilância de contatos de hanseníase, tendo em vista sua construção de forma participativa. Nesse contexto, sua usabilidade é concebida, enquanto TA, como subsídio para o processo de trabalho dos enfermeiros, visando assistência segura e eficaz, bem como facilitadora para comunicação e fortalecendo o trabalho em saúde, principalmente nas ações assistenciais desenvolvidas pelos enfermeiros da APS. Destaca-se a necessidade do investimento em mais pesquisas dessa natureza, visando fortalecer e qualificar o controle da hanseníase, sobretudo o monitoramento dos contatos, na APS.

## Data Availability

Os dados da pesquisa estão disponíveis no corpo do artigo.
